# Prognostic Value and Correlation With Tumor Immune Infiltration of a Novel Metabolism-Related Gene Signature in Pancreatic Cancer

**DOI:** 10.3389/fonc.2021.757791

**Published:** 2022-01-19

**Authors:** Hui Chen, Fuqiang Zu, Taofei Zeng, Ziang Chen, Jinhong Wei, Peng Liu, Zeyu Li, Lei Zhou, Huaitao Wang, Hao Tan, Xiaodong Tan

**Affiliations:** ^1^ General Surgery, Department of Pancreatic and Thyroid Ward, Shengjing Hospital of China Medical University, Shenyang, China; ^2^ General Surgery, Department of Hepatobiliary and Splenic Ward, Shengjing Hospital of China Medical University, Shenyang, China; ^3^ Department of General Surgery, The Second Affiliated Hospital of Harbin Medical University, Harbin, China; ^4^ School of Basic Medical Sciences, Southwest Medical University, Luzhou, China; ^5^ General Surgery, Department of Pancreatic and Endocrine Ward, Shengjing Hospital of China Medical University, Shenyang, China

**Keywords:** pancreatic cancer, metabolism-related genes, prognostic signature, tumor immune microenvironment, immunotherapy

## Abstract

**Background:**

Energy metabolism has been considered as one of the novel features of neoplasms. This study aimed to establish the prognostic signature for pancreatic cancer (PC) based on metabolism-related genes (MRGs).

**Methods:**

We obtained MRGs from the Molecular Signatures Database (MSigDB) and gene sequence data in the Cancer Genome Atlas (TCGA) databases. Then, differentially expressed MRGs (DE-MRGs) were identified utilizing the R software. We built the prognostic model *via* multivariate Cox regression. Moreover, external validation of the prognostic signature was also performed. Nomogram was created to predict the overall survival (OS). Next, this study analyzed the prognostic value, clinical relationship, and metabolism-related signaling pathways of the prognostic signature. The role in tumor infiltration was further evaluated. Eventually, the expression level of the three MRGs along with the function of NT5E was validated.

**Results:**

Twenty-two MRGs were chosen, eight of which were identified to be most significantly correlated with the prognosis of PC. Meanwhile, a 3-MRG prognostic signature was established, and we verified this prognostic model in two separate external cohorts. What is more, the nomogram was used to predict 1-/2-/3-year OS of PC patients. In addition, the immune cell infiltration and expression of immune checkpoint were significantly influenced by the risk score. Finally, three MRGs were highly expressed in PC cell lines, and NT5E was associated with the proliferation and migration ability of PC.

**Conclusion:**

To sum up, the study established and validated a 3-MRG prognostic signature for PC, and the signature could be utilized to predict the prognosis and assist the individualized clinical management of patients with PC.

## Introduction

Pancreatic cancer (PC) remains a rather challenging and devastating disease that seriously threatens human life and health, and recently, published data reveal that it is now the sixth and fourth greatest contributor to death owing to cancer in China and the USA, respectively (1, 2). Although chemotherapy has made great strides in enhancing survival rates, it is dismaying that the 5-year survival rate for PC is only 9%, the lowest of all cancers (2). Therefore, challenges are still existing in the treatment of PC. Given the special anatomical location of the pancreas, PC is commonly symptom-free in the early stage, which only becomes apparent when the surrounding tissues are invaded or distant organs are metastasized (3). In the current case, the exploration of novel prognostic indicators to precisely predict the prognostic outcomes and guide appropriate treatment for patients suffering from cancer, including PC, is considerably urgent (4).

It is well known that tumorigenesis is strongly associated with the activation of oncogenes and the deletion of cancer suppressor genes, which is directly related to alterations in tumor metabolism (5). Otto Warburg discovered that, even in an oxygenated environment, tumor suppressor genes still chose to restrict their energy metabolism primarily to glycolysis, fermenting glucose into lactic acid to meet their energy needs (6). After that, cellular metabolism was believed to be one emerging feature of neoplasms (7). Many metabolism-related pathways are activated and crosstalked in cancer cells to promote cell survival, division, and unlimited growth (8). In addition, the impact of metabolism on the tumor microenvironment (TME) and immune regulation should not be overlooked (9). Altered tumor metabolism leads to the accumulation of specific metabolites in the TME and the increase in extracellular acidification, which facilitates the formation of a pre-metastatic ecological niche and creates a favorable environment for the metastasis of tumors, including PC (5). Therefore, numerous studies have developed novel metabolism-related prognostic signatures for predicting the prognosis of cancer (10–13). Unfortunately, no metabolism-related prognostic model is available to systematically predict the outcome of patients with PC. Thus, the construction of metabolic-based prognostic signatures that can reliably predict the outcomes of PC has remarkable clinical values.

Cancer cells obtain the ability to flourish by disrupting immunosuppressive pathways and using multiple mechanisms, such as recruiting various suppressive immune cells, secreting immunosuppressive cytokines, and producing immunosuppressive metabolites, to induce and enhance immune escape (14, 15). This saves the tumor from being cleared by the organism’s surveillance system, leading to the development of this malignancy from its origin to the abyss. Immunotherapy is an emerging cancer intervention that alters an individual’s immune system to defeat cancer through triggering a direct rejection response or interrupting inhibitory pathways (16). The therapeutic paradigm of targeting immune checkpoint by using monoclonal antibodies to inhibit cytotoxic T-lymphocyte-associated antigen 4 (CTLA-4), programmed cell death protein-1 (PD-1), and programmed cell death protein Ligand-1 (PD-L1) has achieved encouraging results in melanoma, lung cancer, and other malignancies (17). Nevertheless, most phase I and phase II clinical trials failed to demonstrate any clinical benefits on PC (18). The lacking efficacy of routine immunotherapy may be caused by the inherently aggressive biology of PC, abundant demyelinating stroma, poor immunogenicity, and poor infiltration of effector T cells, which pose serious challenges to the success of immunotherapy (19). The hypothesis has been put forward that the future of PC immunotherapy may be combined with chemotherapy, radiotherapy, or co-inhibition of different immune checkpoints (20–22). Of course, whether this will benefit a considerable number of patients with PC demands plenty of in-depth studies to confirm.

The transcriptomic data of common MRGs were utilized to build a prognostic signature, which was validated in two separate external cohorts. Subsequently, nomograms of the predictive model predicting 1-/2-/3-year OS were plotted, and the risk score was an independent prognostic factor for patients of PC. Besides, we operated the Gene Set Enrichment Analysis (GSEA) to find the metabolism-related signaling pathways primarily enriched by the prognostic signature. The study showed distinct differences in the tumor-related immune cell infiltration and immune checkpoints levels within the lower- and upper-risk groups. After that, the expression levels of three MRGs in PC cell lines were confirmed to be upregulated unanimously. Additionally, the NT5E was shown to affect the proliferative and migrative properties of PC cells. In short, this research focused on MRGs to establish one novel metabolism-related prognostic signature in PC, which could be used to guide survival risk stratification of PC to more precisely manage patients with PC.

## Materials and Methods

### Data Collection

The transcriptomic data, simple nucleotide variant (SNV), and matching clinical information for PC were obtained from TCGA (https://portal.gdc.cancer.gov/), GEO (https://www.ncbi.nlm.nih.gov/geo/), and the International Cancer Genome Consortium (ICGC, https://dcc.icgc.org/) database. Then, to increase the comparability and the scientific validity of the TCGA data, the Genotype-Tissue Expression (GTEx) dataset of normal pancreas samples was obtained for differentially expressed analysis on the UCSC Xena (https://xenabrowser.net/) website. Meanwhile, MRGs were extracted from the “c2.cp.kegg.v7.4.symbols” set of metabolic pathway-related genes on the MSigDB (https://www.gsea-msigdb.org/gsea/msigdb/). Subsequently, MRGs that were coexistent in the TCGA and GSE62452 datasets were extracted for subsequent analysis. To keep the gene expression levels in the training and testing sets at the same standard, a log_2_(x + 1) transformation was performed in the datasets from TCGA with ICGC cohort (PACA-AU and PACA-CA) and the datasets from TCGA with GSE57495 for the construction and validation of the prognostic signature (23).

### Enrichment Analysis of MRGs

DE-MRGs in PC compared to normal pancreas tissue were obtained using the R (version 4.0.5) package “limma.” Besides, the threshold for DE-MRGs screening was set to |logFC| > 1, adjusted *p* < 0.05. The volcano map of MRGs in TCGA was plotted using the “ggplot2” package, and the heatmap of MRGs appearing simultaneously in the TCGA and GSE62452 datasets were mapped using the “pheatmap” package. To have a clearer knowledge of the functional properties of the common MRGs and the potential signaling pathways involved, the genes were enriched with Gene Ontology (GO) and Kyoto Encyclopedia of Genes and Genomes (KEGG) utilizing the “clusterProfiler” package.

### Constructing the MRGs Prognostic Signature

Samples with <30 days of follow-up and unclear clinical characteristics were removed to avoid compromising the reliability of this study. One hundred seventy-one PC samples were retained in the TCGA dataset for the construction of the prognostic signature. First, DE-MRGs significantly associated with OS of PC were identified by univariate Cox analysis, setting *p* < 0.01 as statistically significant. The eligible MRGs were applied to the next step of prognostic signature construction. In this study, the least absolute shrinkage and selection operator (LASSO) Cox regression method, running *via* the “glmnet” package, was employed to avoid the prognostic signature overfitting. Then, further multivariate Cox regression analysis of the MRGs detected by the LASSO algorithm was conducted to construct a 3-MRG prognostic signature. For per patient, the risk score is the product of the expression level and the corresponding coefficient of the prognostic signature genes, i.e., risk score = ∑(coefficient_i_* expression of signature gene_i_), coefficient, representing the weight of the corresponding MRG. What is more, we grouped the patients as lower- and upper-risk groups according to median risk values. We used log-rank to test the Kaplan–Meier (KM) survival analysis to compare outcome differences within low- and high-risk groups. Additionally, receiver operating characteristic (ROC) curves were plotted by the “timeROC” package to estimate the predictive accuracy of the prognostic signature.

### Validation of the Prognostic Signature

For validating the universal applicability of this MRGs prognostic signature, two separate external cohorts, the ICGC cohort (PACA-AU and PACA-CA) and the GSE57495 dataset were selected for external validation. The risk score equation from the training cohort was utilized to work out risk scores for individual patients in the validation cohorts. That is to say, individuals from the validation cohorts were classified as low- and high-risk groups according to the threshold of risk values within the training cohort, drawing KM survival curves and ROC curves immediately afterward as described previously.

### Relationship of Risk Score With Clinicopathological Features

This study further verified the expression of three MRGs with the Human Protein Atlas (HPA) online database (https://www.proteinatlas.org/). Next, the univariate and multivariate Cox regression analyses were introduced to determine if this risk score might be related to the prognosis and whether it can act as an independent prognostic factor independently of clinical features (age, gender, histological grade, and pathological stage). Forest plots were drawn with the “forestplot” R package, displaying the *p*-value, HR, and 95% CI for every variable. On the other hand, a nomogram incorporating the risk score with clinical features was constructed using the R package “rms” to predict the 1-/2-/3-year OS of a given patient. The decision curve analysis (DCA) was plotted with “ggDCA” package. In addition, the correlation between the expression of these MRGs together with the risk score and several main clinicopathological features has been explored in subsequent work. Furthermore, time-dependent ROC curves were conducted to assess the prediction performance *via* comparing the risk score with clinicopathological characteristics. In the end, we generated a heatmap indicating the link between the risk score with clinicopathological features and the expression of three MRGs.

### Metabolism-Related Pathways Enriched by Risk Score

Based on mRNA data from 171 PC tissues in the TCGA database, the three MRGs were ranked according to the degree of differential expression in samples from low- and high-risk groups, respectively, using GSEA software (version 4.1.0) to identify potential up- and downregulated metabolism-related signaling pathways. What is more, the sensitivity of PC patients to clinically used chemotherapeutic agents for PC was analyzed using the R software package “pRRophetic.”

### Mutation Landscape Map

The “maftools” R package was utilized to calculate the mutation frequencies of all genes in the SNV data from the TCGA. The comparison of whether the top 20 genes with the highest mutation frequencies were significantly different among the separate risk groups was presented in the form of a waterfall plot. Next, patients with four classical PC driver genes KRAS, CDKN2A, SMAD4, and TP53 (24) wild or mutant type were compared to determine whether their risk scores differed. Aside from this, we compared whether there were differences in tumor mutation burden (TMB) in patients with different clinical characteristics and risks. In the end, the “survminer” package was applied to analyze the survival status of patients with different TMB.

### Risk Score With Tumor Immune Infiltration and Immune Checkpoint Expression

Seven methods including TIMER, CIBERSORT, CIBERSORT-ABS, QUANTISEQ, MCPCOUNTER, XCELL, and EPIC were employed to evaluate cancer immune infiltration to reveal the association between risk score with cancer immune infiltration (25). Pearson correlation analysis was used to evaluate the associations within infiltrating immune cells. The distinctions of immune infiltration cells between the low- and high-risk groups were then compared with the help of the “vioplot” package. Previous studies have suggested that the expression pattern of immune checkpoints may influence the efficacy of immunotherapy (26). We investigated the differentially expressed 47 immune checkpoints like PD-1 (also named PDCD1), PD-L1 (or named CD274), and CTLA-4 in low- and high-risk groups to reveal the potential role of this risk score in immunotherapy response.

### Western Blotting

The steps of Western blotting were carried out as mentioned previously (27). Rabbit antihuman CYP2C18 (ProteinTech Group, Rosemont, IL, USA) and INPP4B (ZEN-Bioscience, Chengdu, China) epitopes polyclonal antibodies and mouse anti-human NT5E and β-actin (ProteinTech Group, Rosemont, IL, USA) epitopes monoclonal antibodies were selected as primary antibodies. Meanwhile, a horseradish-peroxidase-labeled secondary antibody (ProteinTech Group, Rosemont, IL, USA) was used as a secondary antibody for Western blotting.

### Quantitative Real-Time PCR

RNA extraction and quantitative real-time PCR (qRT-PCR) procedures were performed following the description in a prior study (28). PCR primers for CYP2C18, INPP4B, and NT5E were purchased from SangonBiotech (Sangon, Shanghai, China). Primer sequences were shown as follows: CYP2C18, 5′ -GAG TTT TCT GGA AGA GGA A-3′ (forward) and 5′ -GCA TTG GTT TTT CTC AAC TCC T-3′ (reverse); INPP4B, 5′ -GCT GGA TTG GTT TGT GGT TTT A-3′ (forward) and 5′ -TAG CAT TCC AAT TTC ATC GCT G-3′ (reverse); NT5E, 5′ -ACA ACC TGA GAC ACA CAC GGA TG-3′ (forward) and 5′ -TTC GGG AAA GAT CAT ACA CCA CAT GG-3′ (reverse); β-actin, 5′ -CCT GGC ACC CAG CAC AAT-3′ (forward) and 5′ -GGG CCG GAC TCG TCA TAC-3′ (reverse). The expression of CYP2C18, INPP4B, and NT5E was standardized by the internal control β-actin. Meanwhile, fold changes in CYP2C18, INPP4B, and NT5E were calculated in a manner of the 2^−ΔΔCT^.

### Paclitaxel Sensitivity Analysis

Cells were seeded onto 96-well plates, treated with paclitaxel, and incubated for 72 h. After incubation, the CCK-8 reagent (10 μl) was added to each well and incubated for another hour. The absorbance at 450 nm of the color produced in each well was measured with a microplate reader to calculate the number of viable cells. Cells receiving the different treatments were inoculated in 96-well plates. The CCK-8 reagent was added every 24 h, respectively, until 72 h after inoculation and then incubated at 37°C for 1 h before testing the absorbance at 450 nm.

### Cell Transfection

The NT5E shRNA lentiviral vector (hU6-MCS-CBh-gcGFP-IRES-puromycin) was purchased from GeneChem (Genechem Co., Ltd, Shanghai, China). The transfection of Capan-2 and MIA PaCa-2 cells was conducted according to official guidelines.

### Validation *In Vitro*

The colony formation assays were performed to assess the differences in the proliferative capacity of the differently treated cells. The cells from different groups were digested and inoculated in six-well plates (Jet Biofilter Co., Ltd., Guangzhou, China) with 1,000 cells per well. The medium was changed every 3 days and incubated continuously for 10–14 days. After visible colonies were formed, they were immobilized with 4% paraformaldehyde and then stained with crystalline violet (Solarbio Life Sciences, China). To evaluate cellular migration and invasion capacity, the wound healing assays and the Transwell assays were performed. As for wound healing experiments, cells from the different treatment groups were digested and inoculated in six-well plates. A straight line was drawn on the surface of each well with a 100-μl sterile pipette after the cells had grown to approximately 95%. The wound area was photographed using an inverted microscope (Nikon DS-RI2, Japan) at 100× magnification for 0 and 24 h, respectively. For Transwell assays, 800 μl of medium containing 10% fetal bovine serum (Corning, USA) was added to the lower chamber, and 200 μl of serum-free medium containing 20,000 cells was loaded into the upper Matrigel-coated chamber. Cells that had crossed the membrane were fixed with 4% paraformaldehyde after 24 h incubation, washed with phosphate-buffered saline (PBS) and then stained with crystal violet. Images of the cells were taken with an inverted microscope at 200× magnification.

### Animal Experiments

Eight-week-old female BALB/c nude mice (HFK Bioscience Co., Ltd., Beijing, China) were fed in the animal laboratory in a specific pathogen-free level environment. A total of 5 × 10^6^ Capan-2/NT5E knockdown Capan-2 cells were suspended in 300 μl of PBS and injected into the left axilla of the nude mouse. The subcutaneous tumor volume was measured weekly with the formula: V = 0.52 × L × W^2^, where L and W denote the long and short axes of the tumor, respectively.

### Statistical Analysis

The vast majority of statistical analyses and figure outputting was processed with Perl software (version 5.34.0, https://www.perl.org/) and R version 4.0.5 (https://www.r-project.org/). Differential expression of genes was analyzed with the Wilcoxon test, and the two-tailed Student’s t-test was introduced to analyze the relative expression levels of MRGs. The log-rank test was employed for Kaplan–Meier survival curve comparisons. The *p* < 0.05 was accepted as considered statistical significance if not otherwise mentioned. *p < 0.05, **p < 0.01; ***p < 0.001; ns, not significant.

## Results

### Identification of DE-MRGs

**Figure 1:**
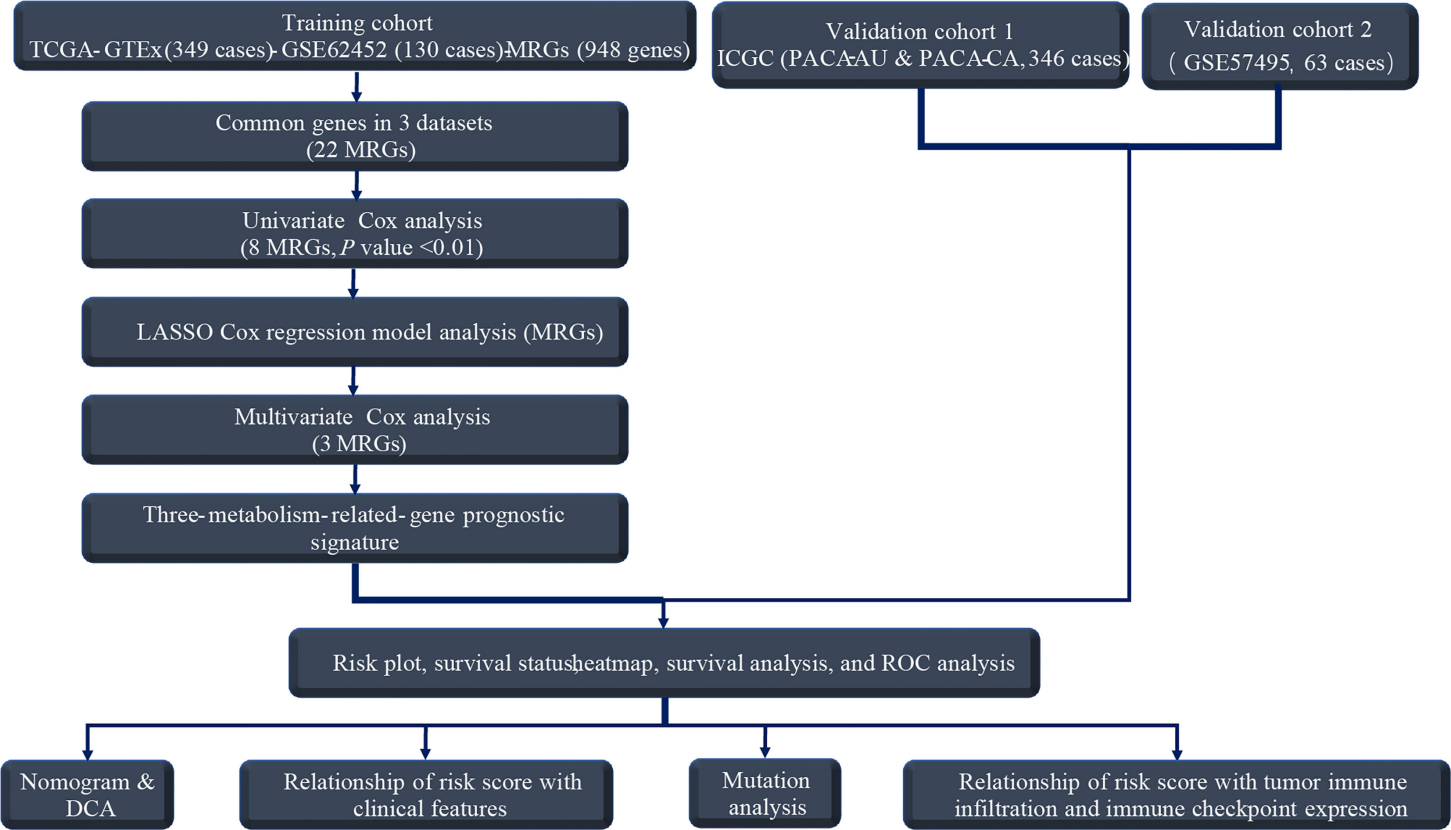
The flowchart for analyzing MRGs-prognostic signature.

**Figure 2:**
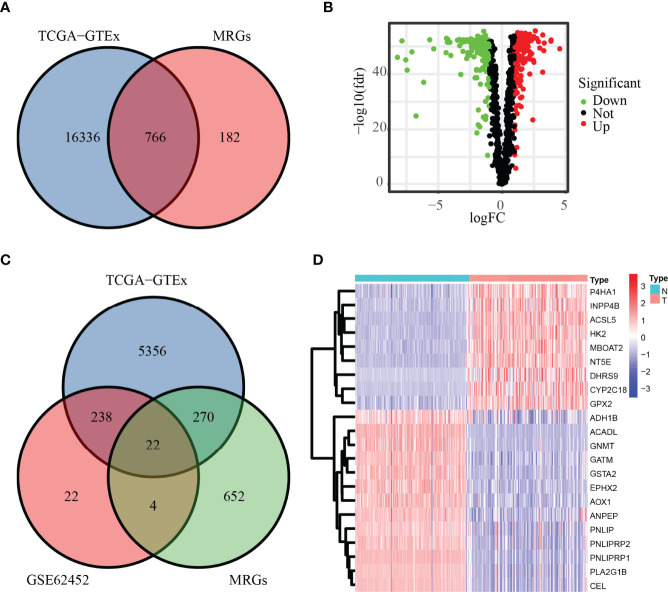
DE-MRGs in PC. **(A, B)** MRGs in the TCGA database are shown in the Venn diagram and volcano plot. **(C)** Common MRGs in the GSE62452 dataset and TCGA database are shown in the Venn diagram. **(D)** Common MRGs are shown in the heatmap.

Our workflow was summarized in the flowchart (**Figure 1**). To determine mRNAs differentially expressed between PC and normal samples, we integrated the datasets of TCGA (178 PC and four normal tissues) and GTEx (167 normal pancreas tissues) as the TCGA-GTEx dataset. The ICGC cohort (PACA-AU and PACA-CA) was a combination of the PACA-AU cohort (262 PC cases) and the PACA-CA cohort (84 PC cases). In addition, two GEO databases were obtained from the GEO database: GSE62452 (69 PC and 61 normal tissues) and GSE57495 (63 PC tissues). Based on the predefined screening criteria (adjust *p* < 0.05 and |logFC| > 1.0), 5,886 genes were significantly differentially expressed in the TCGA-GTEx dataset, consisting of 2,980 overexpressed genes and 2,906 low-expressed genes. Similarly, a total of 286 genes were found in the GSE62452 dataset comprising 188 overexpressed genes and 98 downregulated genes. **Figure 2A** shows that a total of 766 MRGs appeared in the TCGA-GTEx dataset, for which volcano maps were plotted (**Figure 2B**). For another, the metabolism-related pathway gene set contained 948 MRGs, ultimately preserving 22 common MRGs (eight upregulated MRGs and 14 downregulated MRGs) simultaneously existing in all three datasets (**Figure 2C**). The heatmap of the 22 common MRGs is exhibited in **Figure 2D**.

### Functional Enrichment of MRGs

**Figure 3:**
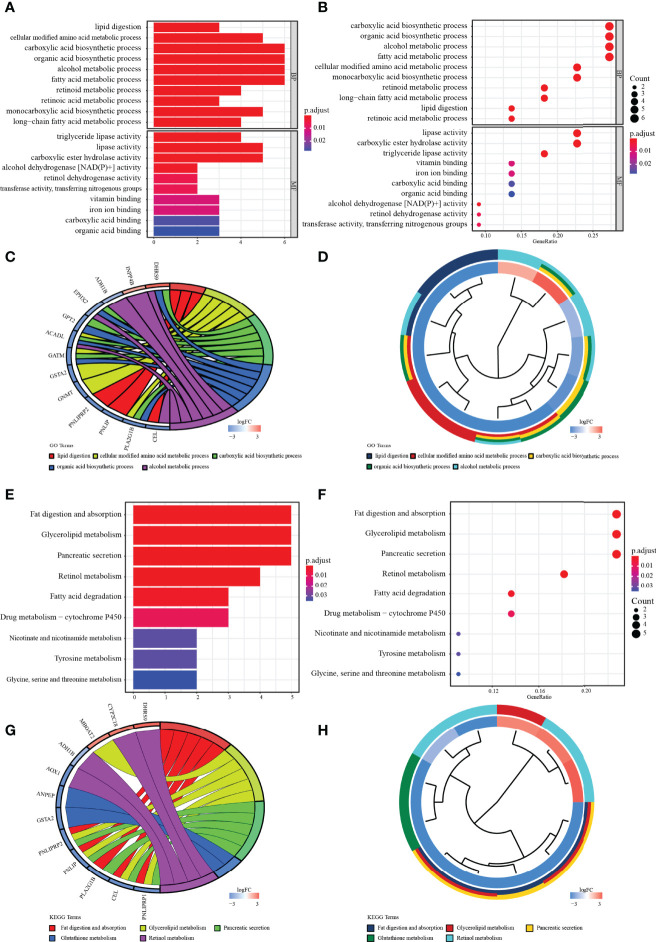
Enrichment analysis. **(A, B)** The bar and bubble charts of GO terms are enriched in BP and MF. **(C, D)** The chord with cluster plots shows the top 5 GO terms and the corresponding enriched genes. **(E, F)** The bar and bubble plots of KEGG enriched terms. **(G, H)** The chord with cluster maps shows the top 3 KEGG terms and the corresponding enriched genes. The false discovery rate (FDR) <0.05 of all terms was significant.

The potential biological functions of the 22 common MRGs were also explored through GO and KEGG enrichment analysis. Altogether, 116 GO terms along with 21 signaling pathways (adjust *p* < 0.05) have entered into our view. Among them, the top enriched GO terms were lipid digestion and triglyceride lipase activity (**Figures 3A, B** **)**. Besides, the top 5 significant GO terms were shown in the chord charts (**Figures 3C, D** **)**. On the other hand, among those enriched KEGG pathways, the common MRGs were strongly related to fat digestion and absorption, glycerolipid metabolism, pancreatic secretion, etc. (**Figures 3E, F** **)**. Meanwhile, the chord diagrams presented the top 5 significantly enriched KEGG signaling pathways (**Figures 3G, H** **)**.

### Establishing the MRGs Prognostic Signature

**Figure 4:**
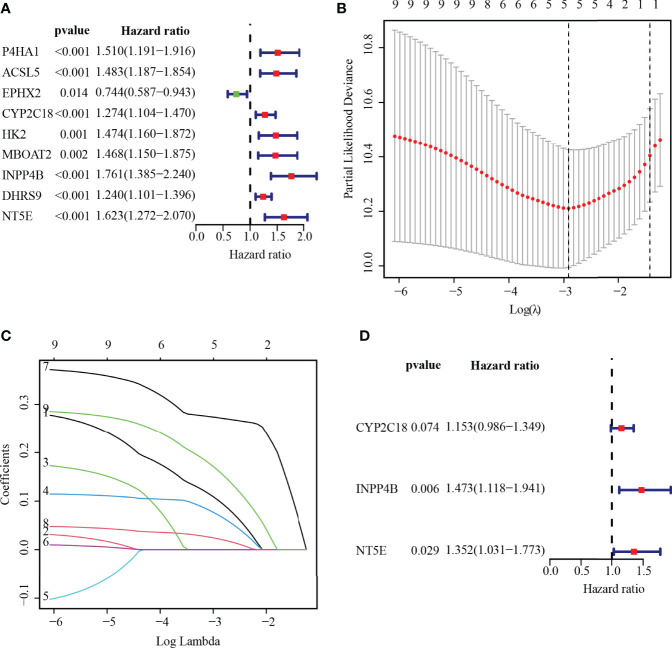
Establishing MRGs prognostic signature. **(A)** Recognition of the outcome-related MRGs in PC *via* univariate Cox regression analysis. *p* < 0.01 were statistically meaningful. **(B)** The tuning parameter (lambda) is determined at the vertical line. **(C)** LASSO coefficient profiles of the 5-MRGs according to the tuning parameter. **(D)** The 3-MRG prognostic signature was built *via* multivariate Cox analysis.

To ascertain the prognostic MRGs, univariate Cox regression analysis was initially applied to analyze the expression matrix and clinical follow-up information of the TCGA cohort for 22 common MRGs. As shown in the results, nine MRGs (P4HA1, ACSL5, EPHX2, CYP2C18, HK2, MBOAT2, INPP4B, DHRS9, and NT5E) were remarkably correlated with the outcomes of PC, among which only EPHX2 was a protective factor for PC (**Figure 4A**; *p*< 0.01). MRG that may be highly intercorrelated with other MRGs was then removed *via* LASSO Cox regression analysis to avoid overfitting, which could confound the prediction results (**Figures 4B, C** **)**. In the end, one prognostic signature was established to assess the prognostic risk for PC patients on the basis of the multivariate Cox regression analysis. As a result, the final metabolism-related prognostic signature was made up of three MRGs: cytochrome P450 family 2 subfamily C member 18 (CYP2C18), inositol polyphosphate-4-phosphatase type II B (INPP4B), and 5′-nucleotidase Ecto (NT5E, or CD73, **Figure 4D**). The predictive model was defined to be one combination of the expression value of the three MRGs weighted by their relative coefficients in the multivariate Cox regression in the following way:


risk score=(0.1426∗expression value of CYP2C18)+(0.3876∗expression value of INPP4B)+(0.3015∗expression value of NT5E)

**Figure 5:**
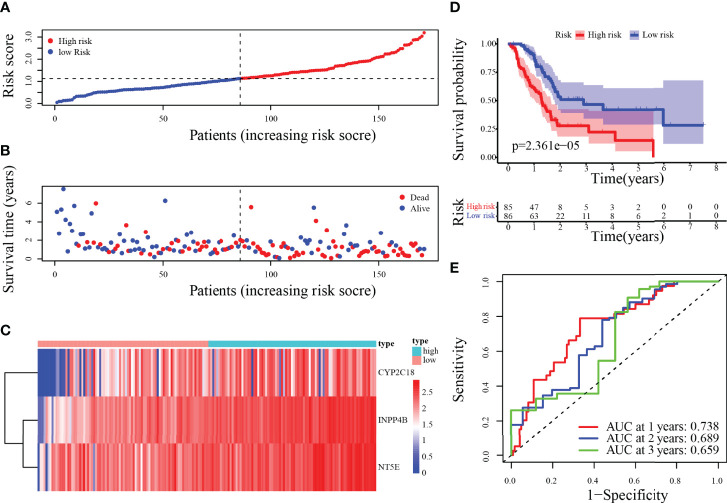
Risk score analysis of the 3-MRG prognostic model in the training cohort. **(A–D)** The risk curve, survival status of PC patients, heatmap of the 3-MRG expression, and survival curves between lower and higher-risk groups are shown. **(E)** Time-independent ROC curves of the risk score for predicting the OS in the training cohort.

The risk score was calculated for each patient according to the prognostic model described above. The 171 patients were categorized by median risk score as a low-risk group (n = 86) and a high-risk group (n = 85). The scatter plots of risk score ranged from lower to higher, and survival status plots are shown in **Figures 5A, B**. Furthermore, the difference in expression of 3-MRG between the low- and high-risk groups was displayed in the heatmap (**Figure 5C**). As indicated in the survival curves, patients from the low-risk group had significantly superior OS to the high-risk group (**Figure 5D**). Besides, the areas under the curve (AUC) values for predicting the 1-, 2-, and 3-year OS were 0.738, 0.689, and 0.659, correspondingly (**Figure 5E**).

### Validation of the Prognostic Signature

**Figure 6:**
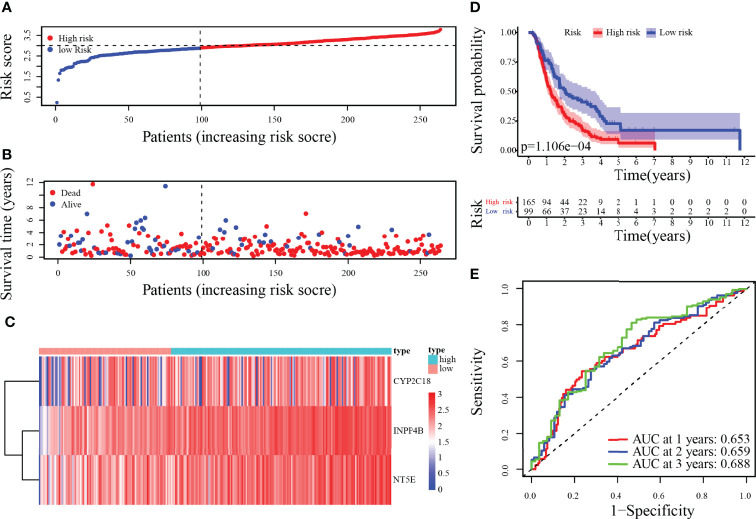
Risk score analysis of 3-MRG prognostic signature in the validation cohort 1. **(A–D)** The risk score, survival status of PC patients, heatmap of the 3-MRG expression, and survival curves between low and high-risk groups are shown. **(E)** Time-independent ROC analysis of risk score for predicting the OS in the ICGC cohort (PACA-AU and PACA-CA).

To validate the metabolism-relevant prognostic signature, the ICGC cohort (PACA-AU and PACA-CA) was used as the validation cohort 1, and the GSE57495 dataset was the validation cohort 2. Patients were then classified as a low- and a high-risk group by the median-risk score of the training cohort. In the validation cohort 1, the risk score, survival status, along with heatmap of 3-MRG have been displayed in **Figures 6A–C**. Concordant with the outcomes generated by the training cohort, the KM curves have revealed that individuals from the higher-risk categories have exceptionally worse outcomes than those in the lower-risk ones (**Figure 6D**). Besides, the predicted AUC values for 1-/2-/3-year OS were 0.653, 0.659, and 0.688, respectively (**Figure 6E**). Similar results in validation cohort 2 are presented in **Supplementary Figures S1A–C**. Overall, the performance of the prognostic signature as a classifier was proved as universally applicable.

### Expression Levels of 3-MRG

**Figure 7:**
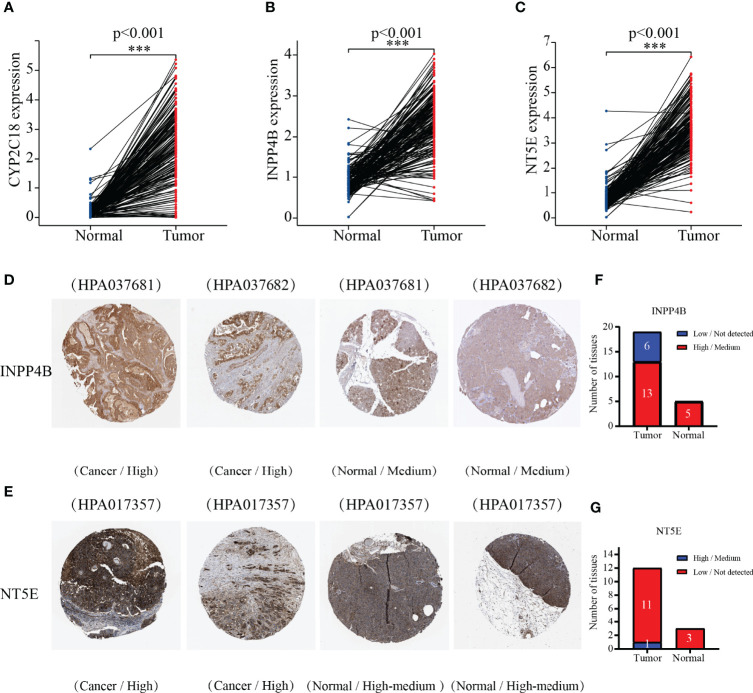
Comparing the 3-MRG mRNA levels in PC and normal pancreas tissues. **(A–C)** CYP2C18, INPP4B, and NT5E. Verifying the 3-MRG expression in PC with normal tissues on the HPA database. Information about CYP2C18 was not available. **(D–G)** INPP4B and NT5E.

We next employed the TCGA-GTEx dataset to analyze the mRNA levels of the 3-MRG. It was shown to be greater in levels of CYP2C18, INPP4B, and NT5E from PC than from normal samples (*p* < 0.001, **Figures 7A–C**). Subsequently, 3-MRG protein levels were assessed on the HPA website. As indicated in **Figure 7D**, the protein level of INPP4B was remarkably greater in PC than in normal tissues. The NT5E was highly expressed in PC tissues along with exocrine glandular cells of normal pancreas tissues and moderately expressed in pancreatic endocrine cells (**Figure 7E**). In PC tissues, INPP4B was highly/moderately expressed in 13 samples and low/non-detected in six samples, while in pancreas tissues, INPP4B was highly/moderately expressed in five samples. In PC tissue, NT5E was highly/moderately expressed in 11 samples and low/non-detected in one sample, whereas it was highly/moderately expressed in three pancreas samples (**Figures 7F, G** **)**. However, the immunohistochemical results of CYP2C18 in PC and adjacent normal tissues were not available.

### Clinical Value of Risk Score

**Figure 8:**
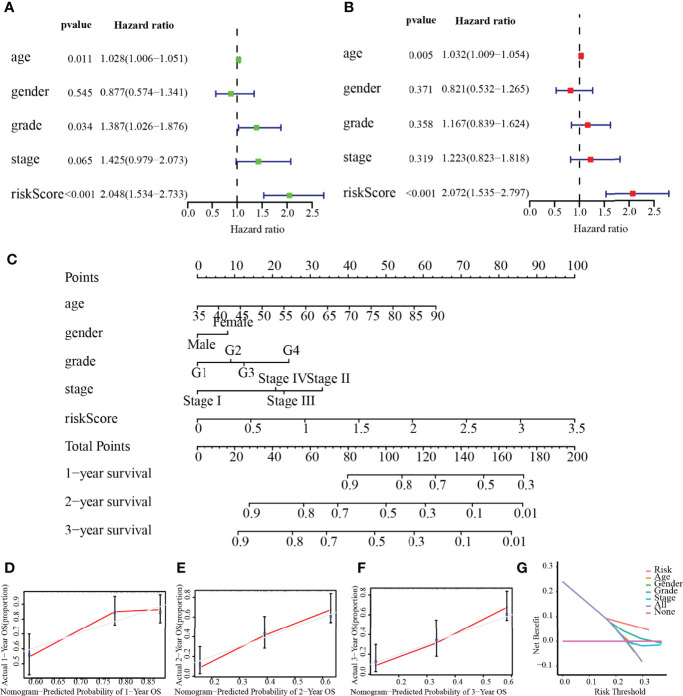
Independent prognostic value of risk score. **(A)** Univariate analysis. **(B)** Multivariate analysis. **(C)** Nomogram based on the signature for PC patients at 1/2/3-year. **(D–F)** Calibration plots of the nomogram at 1, 2, and 3 years. **(G)** DCA of this prognostic signature.

**Figure 9:**
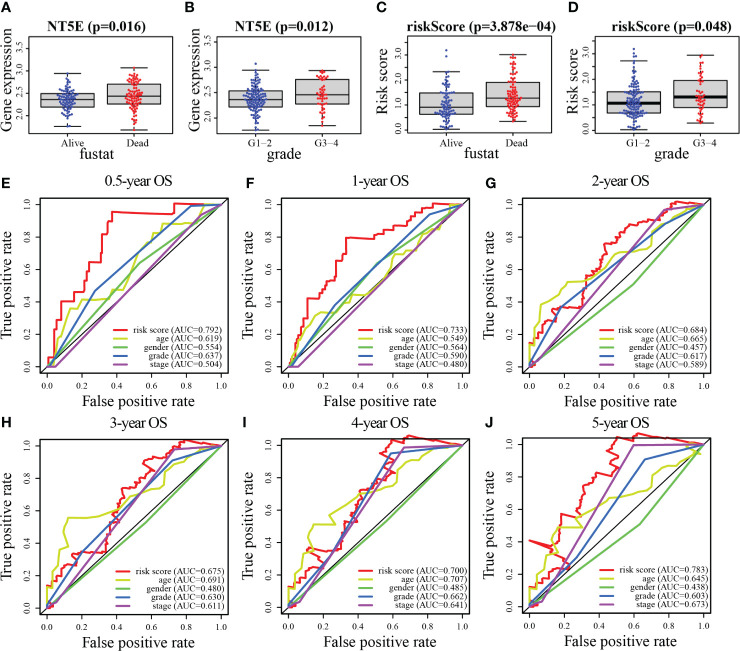
Association between risk score and MRGs expression with clinical features of PC. **(A, B)** NT5E. **(C, D)** Risk score. **(E–J)** The ROC curve analyses of the prognostic variables in the TCGA cohort of 0.5, 1, 2, 3, 4, and 5 years, respectively.

**Figure 10:**
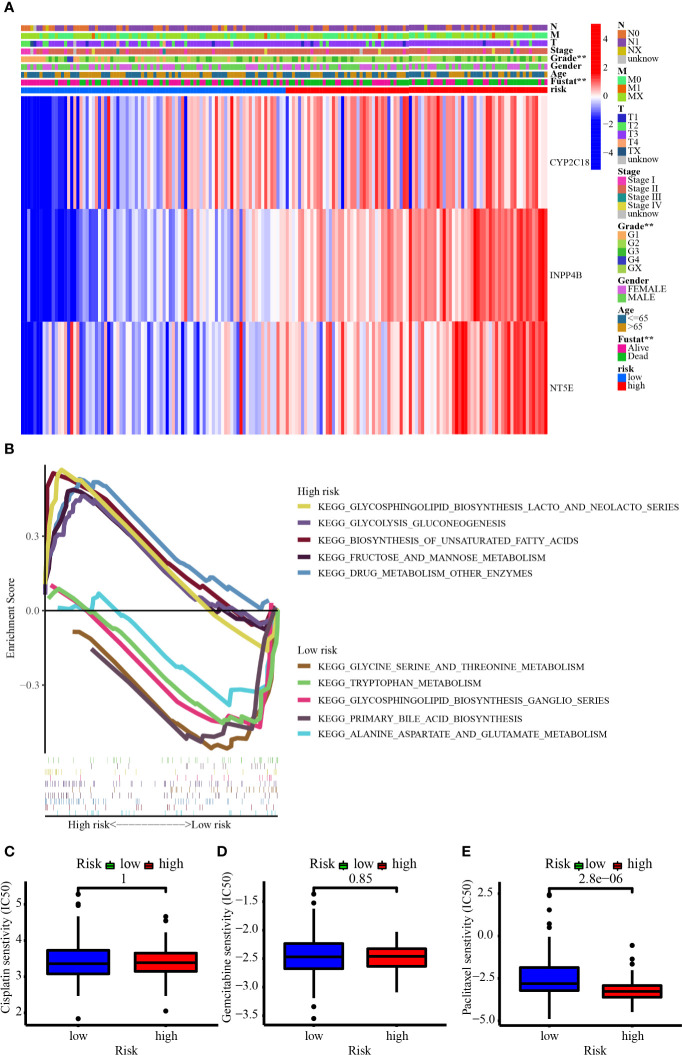
**(A)** The heatmap showed the 3-MRG expression in the prognostic signature based on different risk, clinicopathological characteristics, and survival status in the TCGA cohort. **(B)** GSEA revealed the top 5 up- and downregulated metabolism-related signaling pathways enriched in low- and high-risk groups. **(C–E)** The IC50 showed the chemosensitivity of cisplatin, gemcitabine, and paclitaxel in the low- and high-risk groups.

As depicted in **Figure 8A**, age (*p* = 0.011), histological grade (*p* = 0.034), and risk score (*p* < 0.001) were remarkably associated with the outcomes of patients according to univariate Cox analysis, all of which were high-risk factors for PC. Furthermore, the results of the multivariate analysis suggested that the risk score (*p* < 0.001), and age (*p* = 0.005), can be recognized to be independent prognostic variables (**Figure 8B**). We then plotted the nomogram (C-index = 0.673), which was designed to score patients and predict their OS at 1, 2, and 3 years to appraise the clinical application of the risk score (**Figure 8C**); meanwhile, we have also corrected for the predictive performance of this nomogram, as the results suggested that the predictive performance of the nomogram was satisfactory (**Figures 8D–F**). In addition, the DCA curves showed that the risk score model was superior to other clinical features in predicting clinical benefit (**Figure 8G**). In the next step, the relationship between risk score and MRGs with clinical characteristics was investigated furthermore through assessing the potential clinical application of risk score with MRGs. There was a notable association between NT5E and risk score and clinical factors, where NT5E expression was significantly correlated with survival status (*p* = 0.016) and histological grade (*p* = 0.012, **Figures 9A, B** **)**. Associations between risk score and survival status (*p* = 3.878e−04) along with histological grading (*p* = 0.048) were also significant (**Figures 9C, D** **)**. On the other hand, time-dependent ROC curves were also plotted to investigate other prognostic values of the prognostic signature. The AUC values of 0.792, 0.733, 0.684, 0.675, 0.700, and 0.783 for risk score predicting 0.5-, 1-, 2-, 3-, 4-, and 5-year OS, respectively, were significantly higher than other clinical features (**Figures 9E–J**). The predictive value of this risk score remarkably outperformed other traditional clinicopathological characters. At last, the expression heatmap of the final 3-MRG in the lower- and higher-risk categories and the corresponding clinical information in the TCGA cohort are presented in **Figure 10A**.

### Metabolism-Related Pathways Enriched by Risk Score

**Figure 10B** demonstrates the top 5 up- and downregulated metabolism-related signaling pathways. For one thing, the genes from the high-risk group were largely enriched in the metabolism-related pathways like glycosphingolipid biosynthesis lacto and neolacto series, glycolysis gluconeogenesis, biosynthesis of unsaturated fatty acids, fructose and mannose metabolism, and drug metabolism other enzymes. For another thing, the genes from the low-risk group were largely enriched in the metabolism-related pathways such as glycine serine and threonine metabolism, tryptophan metabolism, glycosphingolipid biosynthesis ganglio series, primary bile acid biosynthesis, and alanine aspartate and glutamate metabolism. Patients in the high-risk group were more enriched in signaling pathways such as glycolysis and lacto biosynthesis, while those in the low-risk group were more enriched in signaling pathways related to amino acid metabolism. **Figures 10C–E** have revealed that patients from the higher-risk group were considerably less sensitive to paclitaxel than those from the lower-risk group, suggesting a horrible outcome in higher-risk individuals may be involved in chemoresistance.

### The Difference in Mutations Between Different Risk Groups

**Figure 11:**
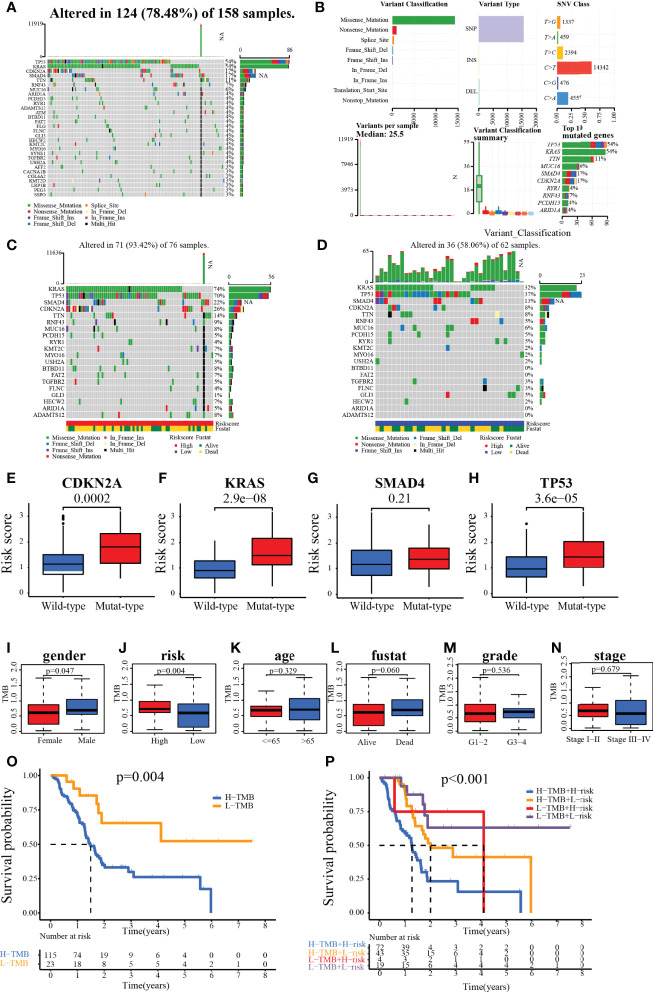
**(A, B)** Waterfall plot for the TCGA patients. **(C, D)** Waterfall plot for the patients in the high- and low-risk group. **(E–H)** Relationship between genes (CDKN2A, KRAS, SMAD4, and TP53) status and risk score. **(I–N)** The association of clinical characteristics and risk scores with TMB. **(O, P)** Kaplan–Meier survival analysis of patients with different TMB with/or different risks.

Analysis of the SNV data from TCGA showed that a total of 124 of the 158 samples harbored significant mutations. The most frequent mutations were in the driver genes of PC (TP53, KRAS, CDKN2A, and SMAD4), and the most common variant classification was the missense mutation (**Figures 11A, B** **)**. In the high-risk group, 56 patients harbored KRAS mutations, 53 patients carried TP53 mutations, 17 patients had SMAD4 mutations, and 20 patients harbored CDKN2A mutations. In the low-risk group, 20 patients had KRAS mutations, 23 patients harbored TP53 mutations, 8 patients carried SMAD4 mutations, and 5 patients harbored CDKN2A mutations (**Figures 11C, D** **)**. Further analysis revealed that patients with mutated KRAS, CDKN2A, and TP53 presented significantly higher risk scores than those with wild type of the relevant genes (**Figures 11E–H**). We also found that TMB was significantly higher in male patients or patients with high risk (**Figures 11I–N**). Patients carrying H-TMB had a significantly shorter survival time than those with L-TMB. Survival analysis combining the risk and TMB subgroups revealed that high-risk patients harboring H-TMB had the worst prognosis, those at low risk of having H-TMB showed the second-worst prognosis, and those at low risk of having L-TMB presented the best prognosis (**Figures 11O, P** **)**.

### Risk Score With Tumor Immune Infiltration and Immune Checkpoint Expression

**Figure 12:**
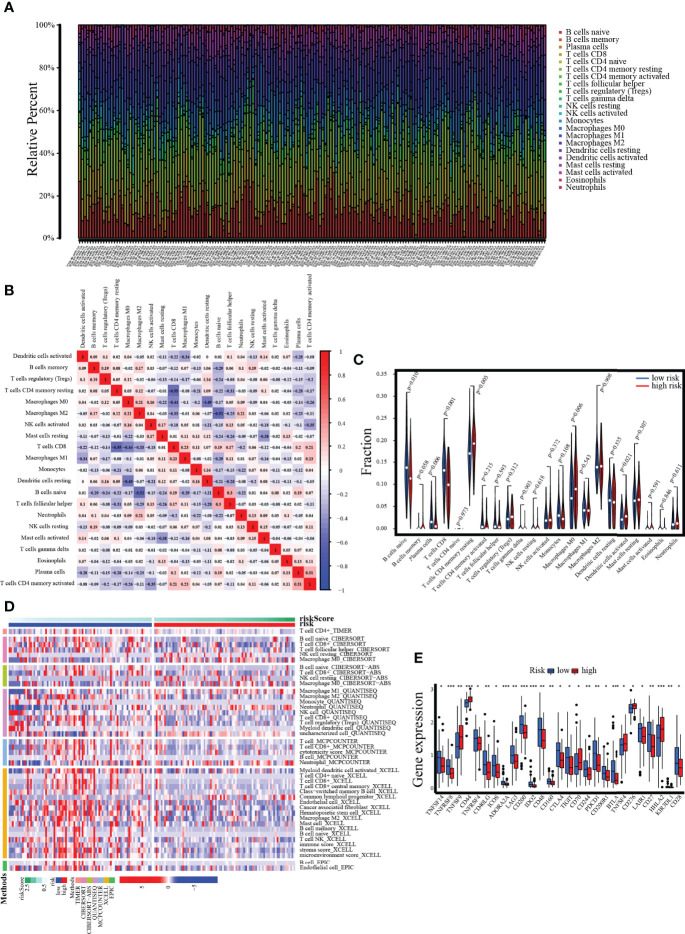
Assessment of tumor-infiltrating cells and immunotherapy value. **(A)** The bar chart reveals the percentage of 22 infiltrating immune cells from the TCGA cohort. **(B)** The correlation matrix shows correlations between infiltrating immune cells in tumor samples. **(C, D)** Violin plot and heatmap present the differentially infiltrated immune cells in lower- and higher-risk groups. **(E)** The differential expression of immune checkpoints between low- and high-risk groups.

In the PC sample of the TCGA dataset, the 22 immune cell infiltration landscapes with a marked difference in the percentage of immunocytes among samples are observed in **Figure 12A**. The correlation matrix showed the relationships between different infiltrating immune cells in tumor samples. From the correlation heatmap, the proportion of 22 infiltrating immune cells was correlated gradually. T cells CD8 was most strongly negatively correlated with T cells CD4 memory resting (Pearson’s correlation = −0.55), and B cells naive was moderately negatively correlated with macrophages M2 (Pearson’s correlation = −0.52), whereas T cells CD4 memory activated showed the strongest positive correlation with plasma cells (Pearson’s correlation = 0.31, **Figure 12B**). In addition, the results of the violin plot revealed that the low-risk group included notably higher numbers of B-cell naive, plasma cell, and T-cell CD8 infiltrates, while the high-risk group had comparably higher numbers of T-cell CD4 memory resting, dendritic cells activated, etc. (**Figure 12C**). Next, a heatmap of the proportions of the 22 immune cell types showed that there was an increased number of infiltrations of macrophage M0 and NK cells resting in the high-risk group, while the immune score and stroma score, along with microenvironment score, were lower, whereas the infiltrating number of B-cell, T-cell, and cytotoxicity score was higher in the low-risk group (**Figure 12D**). On the other hand, the immune checkpoints with low expression in the high-risk group were PDCD1, CTLA4, and CD28. Conversely, TNFSF9, CD44, CD70, TNFSF4, CD276, and HHLA2 were overexpressed in the high-risk group (**Figure 12E**).

### Experimental Validation

**Figure 13:**
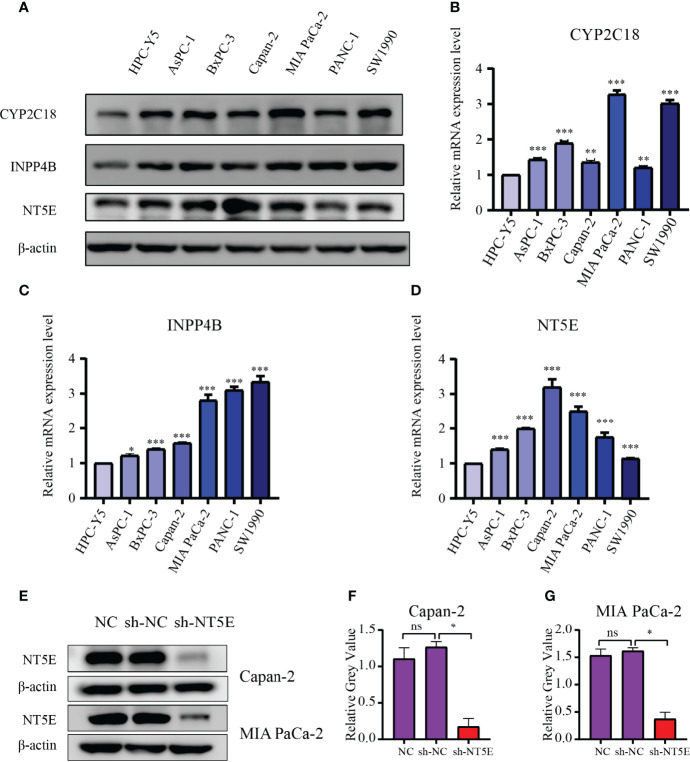
Validation of the 3-MRGs levels *via* WB and qRT-PCR. **(A)** The protein levels of the three MRGs in six PC cell lines and pancreatic epithelial cells. **(B–D)** The mRNA expression of the three MRGs in six PC cell lines and pancreatic epithelial cells. **(E–G)** Confirmation of NT5E knockdown efficiency by WB. *p < 0.05, **p < 0.01, ***p < 0.001, ns, not significant.

**Figure 14:**
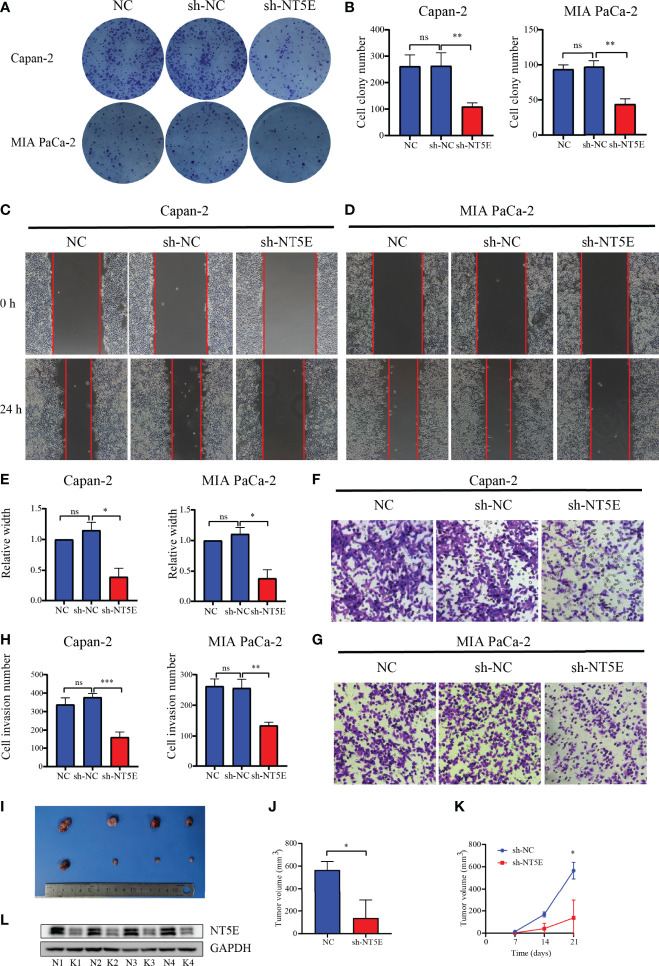
Validation of the function of NT5E *via* colony formation assay, wound-healing assay, Transwell assay, and animal experiments. **(A, B)** Colony formation assays. **(C–E)** Wound-healing assays. **(F–H)** Transwell assays. **(I–K)** Animal experiments. **(L)** Confirmation of NT5E knockdown efficiency *in vivo* by WB. N, negatively control; K, NT5E knockdown. *p < 0.05, **p < 0.01, ***p < 0.001, ns, not significant.

The CYP2C18, INPP4B, and NT5E expression levels were examined in six PC cell lines (AsPC-1, BxPC-3, Capan-2, MIA PaCa-2, PANC-1, and SW1990) and normal pancreas epithelial cells (HPC-Y5) was used as control. The levels of CYP2C18, INPP4B, and NT5E were notably higher in six PC cell lines than in the HPC-Y5 cell line (**Figure 13A**). On the other hand, similar results were observed at the mRNA level (**Figures 13B–D**). Previously, the NT5E was reported to be associated with the prognosis of PC (29). Hence, we knocked down NT5E expression to assess the effect of NT5E on the biological behavior of pancreatic cancer. Western blotting was performed to detect NT5E knockdown efficiency (**Figures 13E–G**). Colony formation assays revealed that the proliferative capacity of Capan-2 and MIA PaCa-2 cell lines was noticeably suppressed after NT5E knockdown (**Figures 14A, B** **)**. Wound healing assays indicated that knockdown of the NT5E gene resulted in a significant reduction in migration of Capan-2 and MIA PaCa-2 cell lines (**Figures 14C–E**). Moreover, Transwell assays demonstrated that the invasive ability of Capan-2 and MIA PaCa-2 cell lines was also dramatically restrained upon NT5E knockdown (**Figures 14F–H**). The subcutaneous xenograft model was constructed to confirm the effect of NT5E on PC growth *in vivo*. NT5E knockdown inhibited the growth of xenograft tumor *in vivo* (**Figures 14I–K**), and Western blotting detected the relative expression of NT5E (**Figure 14L**). The above results suggest that NT5E may be an important promoting factor for PC. As demonstrated by drug sensitivity assays, enhanced sensitivity of Capan-2 and MIA PaCa-2 cell lines to paclitaxel could be observed after knockdown of NT5E, which indicated that the signature gene NT5E was involved in mediating resistance to paclitaxel in PC (**Supplementary Figures S2A–D**).

## Discussion

As of today, pancreatic cancer remains a very disruptive malignancy at the global level with a rather poor prognosis. An accurate prediction of OS in PC would facilitate the personalized treatment regimen. However, nearly no reliable and valid biomarkers to predict the prognosis of PC are currently available, for which the need to identify credible biomarkers and predictor patterns to foresee the outcome of PC is urgent. In this study, we built and verified a novel prognostic signature with MRGs based on outcome forecasting in PC patients.

To explore the value of metabolism-related genes for prognosis prediction and treatment guidance in PC. Two research teams have now established a metabolism-related prognostic signature in PC. Huo et al. (30) developed a prognostic signature based on 15 MRGs to predict the prognosis of PC patients. The proportion of CD8+ T cells infiltrated was higher in low-risk patients from this model, which predicted longer survival. Furthermore, the expression of PD-L1 was positively correlated with the risk score, whereas high PD-L1 expression induced immune escape and a worse prognosis. However, the authors did not conduct further studies on the expression of numerous immune checkpoints to explore whether risk stratification is a guide to immunosuppressive therapy. Tan’s work (31) was more concerned with metabolic reprogramming, which is one of the hallmarks of cancer, including enhanced lipid metabolism and glycolysis (32, 33), and therefore established a three-gene prognostic signature based on genes related to glycolysis and lipid metabolism. The clinical therapeutic value of targeting the signature gene c-Met for oncogenesis and metastasis and the association of CD36 with PC resistance to gemcitabine were proposed. Unfortunately, the study did not further stratify the patients with the help of the prognostic signature to more precisely indicate the patients benefiting from c-MET-targeted therapy or the patients resistant to gemcitabine. Finally, the signatures established by both teams were based on bioinformatic level analysis with no further exploration of the impact of signature genes on the biological behavior of PC.

In the current study, a 3-MRG prognostic signature was established using LASSO Cox and multifactorial Cox regression, which could differentiate between PC patients with different risks. The risk score was an independent prognostic factor for PC, and a nomogram containing clinical features was constructed on this basis. Given that paclitaxel occupies a very critical role in the clinical management of PC, the sensitivity to paclitaxel was lower in high-risk patients, who had higher expression of NT5E. NT5E gene silencing can dramatically enhance paclitaxel sensitivity in Capan-2 and MIA PaCa-2 cells. It was hypothesized that NT5E may be involved in mediating chemoresistance in high-risk patients. According to our prognostic model, patients in the low-risk group may benefit more from treatment with paclitaxel than those in the high-risk group. One application of our model was to provide a theoretical basis to guide clinical decisions on chemotherapy regimens for patients with PC. This is a complement of this research to those two relevant publications in terms of conceptual and practical aspects. In the high-risk group, the frequency of mutations in PC driver genes was dramatically higher than those in the low-risk group. High risk was also associated with high TMB, where patients at high risk with high TMB exhibited the worst outcomes. There were considerable differences in the abundance of tumor-infiltrating immune cells and in the expression of the immune checkpoint profile among different risk groups. Ultimately, the biological role of the signature gene NT5E in PC was rigorously validated by experiments *in vitro* and *in vivo*, which took this study a step forward, which is what this study exceeds these two relevant publications in practical advance.

In recent years, a substantial number of studies have been devoted to the identification of molecular subtypes of tumors, and NT5E was found to be differentially expressed in different subtypes of PC. In Collison’s work (34), the NT5E gene was found to be upregulated in a highly aggressive quasi-mesenchymal subtype. Similarly, Bailey et al. (35) revealed that NT5E gene expression was upregulated in gene programs 2 (GP2) of the mutant TP53 knockout squamous cell subtype. The two subtypes refer to the same PC subtype (quasi-mesenchymal and squamous cell subtype) as defined by the TCGA pan-cancer analysis (35). Other metabolic subtyping/stratification studies have also yielded associations between a glycolytic metabolism in PDAC with mesenchymal features and poor prognosis, where a lipogenic phenotype is associated with epithelial features and better prognosis (36). More recently, the work by Gao et al. has identified that INPP4B was enriched in a metabolic gene enriched subtype (C1) and was more highly expressed in patients at high risk of this subtype, which had a worse prognosis (37). There is an association between human cytochrome P450 (CYP) enzyme activity with the risk of multiple cancers. However, the correlation between CYP2C18 and cancer risk has not been reported (38).

Overall, KEGG enrichment suggested that all PC patients in TCGA were predominantly enriched in pathways associated with lipogenic metabolism, which was relevant to epithelial (classical) subtype characteristics with better prognostic (36, 39). Nevertheless, there is pronounced metabolic heterogeneity in PC patients, and enrichment analysis in general for all patients may not elicit accurate conclusions (40). Therefore, patients may benefit more from further patients subtyping or stratification to locate patients with similar metabolic patterns and target their metabolic characteristics. With further risk stratification of patients, a shift in metabolic pattern towards glycolysis was noted in high-risk patients, accompanied by increased lactate production. This conversion was accompanied by an enrichment of the glycolysis-related genes LDHA and ENO_2_, which are responsible for catalyzing the final step of aerobic glycolysis, facilitating the glycolytic process by transforming pyruvate into lactate (41). Acidification of the extracellular microenvironment through increasing lactate production promotes tumor invasion and metastasis (42). Such patients were mainly tightly related to the mesenchymal (QM-PDA) subtype, showing a more aggressive capability and markedly worse prognosis (36, 39). Various preclinical studies have shown that blocking LDHA to reduce glycolysis could inhibit tumor growth and metastasis, presumably indicating LDHA as a potential therapeutic target (43–46). In Rajeshkumar’s work, treatment of pancreatic cancer cells carrying mutated TP53 with FX11, a small molecule inhibitor of LDHA, was revealed to have decreased metabolic activity, increased apoptosis, and attenuated tumor growth (42). In the high-risk group, the tumors were primarily glycolytic, carrying a higher proportion of TP53 mutation frequencies, which may imply a potential metabolic vulnerability to glycolytic inhibitors (e.g., FX11) in this group of patients. This is a further confirmation of the value of our model for risk stratifying patients for treatment guidance.

Cancer is caused by the accumulation of somatic mutations in oncogenes and tumor suppressor genes (47). KRAS mediates downstream signaling of growth factor receptors, and its mutational activation drives over 90% of pancreatic cancers (48). Oncogenic mutations in KRAS are involved in regulating alterations of cell metabolism, mainly in the form of increased uptake of glucose, a shift from oxidative phosphorylation to aerobic glycolysis, and enhanced levels of lactate (49, 50). All these alterations would result in enhanced proliferation, migration, and invasion of PC cells (51, 52). This study indicated that more KRAS mutations were carried by patients from the high-risk group who were more likely to exhibit aggressive metastasis. Liang and colleagues demonstrated that SMAD4 mutations induce upregulation of phosphoglycerate kinase 1 (PgK1) expression in PC, the first ATP-generating enzyme in the glycolytic pathway (53). Nuclear PgK1 preferentially drives cell metastasis *via* mitochondrial oxidative phosphorylation (54). An earlier study by Oshima suggested that mutations in SMAD4 were significantly associated with tumor size, lymphatic infiltration, and lymph node metastasis, whereas CDKN2A mutations were associated with lymphatic infiltration and extensive postoperative metastasis. High-risk patients screened by our prognostic model had a higher frequency of SMAD4 and CDKN2A mutations (although the former were not significant), suggesting that this group of patients may be more susceptible to lymphatic infiltration or metastasis and extensive postoperative recurrence. Studies have indicated that TP53 inactivation was associated with the malignant progression of PC (35, 55). In another study, Morton and colleagues revealed that only TP53 mutant PC cells exhibited invasive activity compared to TP53 gene deletion (56). In conclusion, mutations in genes may allow cancer cells to acquire more aggressive properties by altering their metabolic pattern.

A correlation between infiltrating immune cells in the TME with the prognosis of many cancers (e.g., ovarian cancer, kidney cancer, colorectal cancer, breast cancer, and pancreatic cancer) has been increasingly observed (30, 57). The CD8+ and CD4+ T cells, dendritic cells (DCs), regulatory T cells (Treg), and tumor-associated macrophages (TAMs) are mainly present in TME (58). It is generally accepted that the presence of T-cell infiltration predicts a better prognosis (59, 60). This study also identified significantly lower infiltration of CD8+ T cells in high-risk patients, consistently predicting a worse prognosis for these patients. Studies have suggested that T cells entering a resting state would reduce the proliferation of CD8+ T cells in lymph nodes, preventing them from recognizing cancer cells, which effectively mitigated the body’s immune response and promoted further proliferation of cancer cells (61, 62). In the high-risk group, the proportion of CD4+ T-cells memory resting was dramatically higher, possibly reducing the proliferation of CD8+ T cells, which prevented the organism’s immune system from recognizing the cancer cells and producing an effective immune response. In this case, the cancer cells were permitted to continuously proliferate. In addition, the TAMs were suggested to be associated with poor prognosis and rapid disease progression (63, 64). Xu et al. (65) proposed that M0 macrophages were an independent predictor of poor prognosis in patients with PC after finding an accumulation of M0 macrophages in the tumor tissue. The prognostic value of M0 macrophage infiltration was further confirmed by the current study, where the level of M0 macrophage infiltration was markedly higher in high-risk patients with a worse prognosis. Besides, dendritic cells activated in this study exhibited a greater level of infiltration in the high-risk group, which may facilitate the metastasis and immune escape of cancer cells by upregulating the immunosuppressive WNT pathway (65, 66). Overall, the infiltration levels of several immune cells related to the prognosis of PC patients differed significantly in the current study, and the TME may act on tumor metastasis and immune escape through these immune cells. The identification of genes aberrantly expressed in tumors is important for the development of individualized therapies, which could improve treatment outcomes (67). The expression levels of PD-1 and CTLA4 are highly correlated with the prognosis of patients receiving immunotherapy. Liu et al. revealed that high serum PD-1 and CTLA4 levels predicted better outcomes for LIHC patients treated with cytokine-induced killer cell (CIK) immunotherapy (68). PD-1 was highly expressed in older cancer patients. Similarly, PD-1 and CTLA4 levels were higher in black patients than in Caucasian or Asian patients, who may have had better outcomes with immunotherapy (68). The combination of anti-PD-1 and CTLA4 immune checkpoint inhibitors in several cancers demonstrated better efficacy than monotherapy (69–72). Analysis of immune checkpoint expression profiles revealed that PD-1 and -CTLA4 had higher expression levels in the low-risk group of patients, inferring therapeutic opportunities for novel immune modulators in low-risk patients. This result also highlighted the importance of our model in guiding the clinical personalized treatment of patients.

Admittedly, a few limitations exist during the current work. Initially, our prognostic model was based on mRNA expression levels for predicting patient prognosis, but the protein levels of genes may be more closely aligned with the real conditions of patients in actual clinical situations. Validation of the association between protein expression levels of signature genes with patient prognosis is currently not achievable due to the unavailability of clinical specimens and corresponding survival information; therefore, this very critical component will need to be refined in later clinical work. Second, the reliability and stability of our prognostic signature have to be proven on a large PC research center with prospective studies. Third, the bio-functions of the other two MRGs in PC remain to be further verified through a series of experiments.

## Conclusion

A novel prognostic signature based on 3-MRG and a prognostic nomogram to predict 1-/2-/3-year OS for PC. Molecular features based on this signature offered fresh perspectives on the malignant development of PC. The difference between immune cell infiltration and the level of immune checkpoint expression in PC could be an important guide to the prognosis and treatment of PC patients. NT5E is of crucial importance for the malignant behavior of pancreatic cancer. This prognostic signature may be of great value in the prognostic assessment and treatment guidance for PC patients.

## Data Availability Statement

The original contributions presented in the study are included in the article/**Supplementary Material**. Further inquiries can be directed to the corresponding author.

## Ethics Statement

The animal study was reviewed and approved by the ethics committee of the Affiliated Shengjing Hospital of China Medical University.

## Author Contributions

HC designed the project under the direction of XT. HC, FZ, and TZ collected data and wrote the first draft. HC, TZ, ZC, JW, PL, and ZL did the statistical analysis and output the figures. HC and FZ performed the validation experiments. LZ, HW, and HT did the interpretation of data for the work and helped with part of English writing. All authors contributed to the article and approved the submitted version.

## Funding

This research was supported by the evaluation of the perioperative curative effect of main pancreatic duct jejunum bridge catheter drainage in robotic pancreaticoduodenectomy (grant number 2020JH 2/10300130) and clinical application and promotion of a novel early diagnosis method for pancreatic cancer (grant number 2020JH6/10500055).

## Conflict of Interest

The authors declare that the research was conducted in the absence of any commercial or financial relationships that could be construed as a potential conflict of interest.

## Publisher’s Note

All claims expressed in this article are solely those of the authors and do not necessarily represent those of their affiliated organizations, or those of the publisher, the editors and the reviewers. Any product that may be evaluated in this article, or claim that may be made by its manufacturer, is not guaranteed or endorsed by the publisher.

## Abbreviations

TCGA, The Cancer Genome Atlas; GEO, Gene Expression Omnibus; GTEx, Genotype-Tissue Expression; MsigDB, Molecular Signatures Database; ICGC, International Cancer Genome Consortium; PC, pancreatic cancer; MRGs, metabolism-related genes; DE-MRGs, differentially expressed metabolism-related genes; FDR, false discovery rate; GO, Gene Ontology; KEGG, Kyoto Encyclopedia of Genes and Genomes; KM, Kaplan–Meier; OS, overall survival; ROC, receiver operating characteristic; AUC, area under the curve; HR, hazard ratio; CI, confidence interval; SDS-PAGE, sodium dodecyl sulfate–polyacrylamide gel electrophoresis; PVDF, polyvinylidene fluoride; TME, tumor microenvironment.
