# Supplementary material for: Prognostic Value and Correlation With Tumor Immune Infiltration of a Novel Metabolism-Related Gene Signature in Pancreatic Cancer
Source: Front Oncol. 2022 Jan 19;11:757791. doi: 10.3389/fonc.2021.757791 (PMC8807690; doi:10.3389/fonc.2021.757791)
Supplement: Supplementary file 1 [file DataSheet_1.docx]

Supplementary Material

## Supplementary Figure1


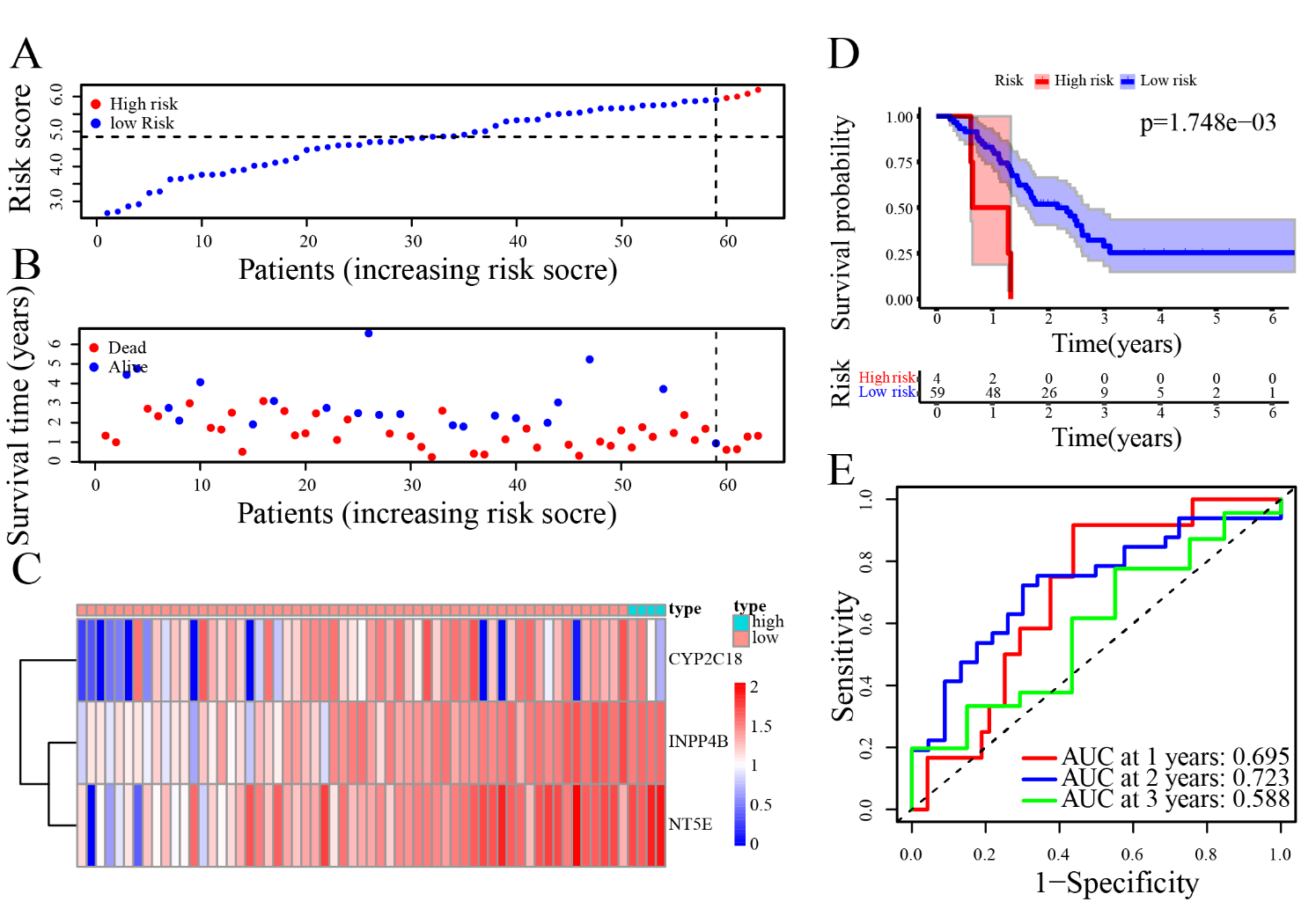


**Supplementary Figure 1.** Risk score analysis of 3-MRG prognostic signature in the validation cohort 2. (A-D) The risk score, survival status of PC patients, heatmap of the 3-MRG expression, and survival curves between low and high-risk groups were shown. (E) Time-independent ROC analysis of risk score for predicting the OS in the GSE57495 dataset.

## Supplementary Figure2


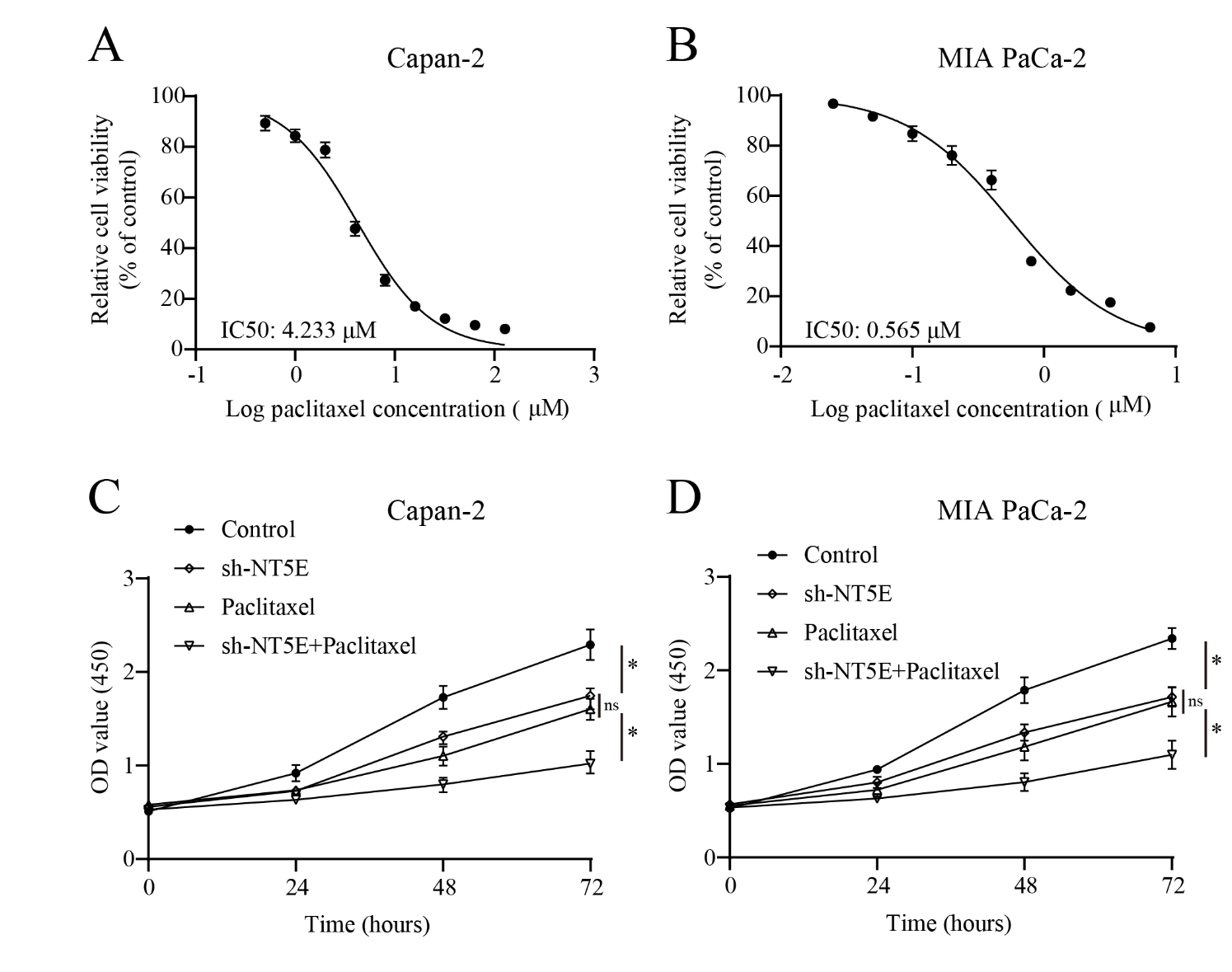


**Supplementary Figure 2.** Knockdown of NT5E increased chemosensitivity to paclitaxel in Capan-2 and MIA PaCa-2 cells. (A-B) Sensitivity of Capan-2 and MIA PaCa-2 cells to paclitaxel. (C-D) Growth curves of Capan-2 and MIA PaCa-2 cells receiving different treatments.
